# CUSTODIAL AND CO-RESIDENT GRANDMOTHERS SERVICE USE AND NEEDS: 10 YEARS APART

**DOI:** 10.1093/geroni/igz038.1843

**Published:** 2019-11-08

**Authors:** Alexandra B Jeanblanc, McKenzie Wallace, Carol M Musil

**Affiliations:** Case Western Reserve University, Cleveland, Ohio, United States

## Abstract

The period between 2008 and 2017 spans a period of economic and societal flux in America, including the 2008 recession, the Affordable Care Act, and the emergence of the opioid crisis. These changes have had profound effects on families, including the rise in grandparent-headed and multigenerational households and their financial, resource, and service needs. We compared the service needs and utilization patterns of 334 co-resident grandmothers who were participants in two independent NIH-funded studies of grandmothers raising grandchildren, conducted 10 years apart (2008-2009:n=145 and 2018-2019:n=189) Researchers asked both samples whether they received or had an unmet need for 16 types of services (ex: financial, legal, substance abuse, respite care). There were statistically significant differences between the samples on 8 of 16 services received, and 15 of 16 unmet, but recognized, service needs, with our current sample reporting higher usage and need. The largest percentage increases in need were financial assistance (31.5%), respite care (29.9%), exercise/fitness (26.4%), and family therapy (25%). Although there is a 7% increase in legal services received between the samples, 48.1% of the 2018-19 sample still report unmet legal needs, compared to 16.6% of the 2008-09 sample. We observed a significant difference in an unmet need for help to families dealing with drug and alcohol abuse, with 23% of current participants reporting unmet need, compared to 9% in the prior study. Our results highlight a gap in resources available to grandfamilies and highlight the need for intervention and evaluation research targeting service needs to inform policy change.

